# Comparison of the Diagnostic and Therapeutic Efficacies of Portable Recruited Chest Radiography with Conventional Portable Radiography in Mechanically Ventilated Patients

**Published:** 2019-04

**Authors:** Ali Sharifpour, Abdulrasool Alaee, Masoud Aliyali, Siavash Abedi, Neda Karimi

**Affiliations:** 1 Pulmonary and Critical Care Division, Mazandaran University of Medical Sciences, Sari, Iran; 2 Department of Radiology, Mazandaran University of Medical Sciences, Sari, Iran.

**Keywords:** Portable chest radiography, CXR, Recruited chest radiography, Mechanical ventilation, ICU

## Abstract

**Background:**

In mechanically ventilated patients, portable chest radiography (CXR) can provide important information for selecting the optimal therapeutic approach. This study aimed to determine the diagnostic and therapeutic efficacies of portable recruited chest radiography with maximum inspiratory volume and pause in comparison with conventional portable radiography.

**Materials and Methods:**

This diagnostic accuracy study was conducted on 75 mechanically ventilated patients admitted to the intensive care unit (ICU) of Imam Khomeini Hospital in Sari, Iran, during 2013–2015. For every patient, in addition to conventional portable CXR, another CXR was performed with mechanical ventilator adjustments (tidal volume up to 10–12 ml/kg to maintain the inspiratory plateau pressure below 35 cmH_2_O and inspiratory time of 2–3 seconds). CXR was performed after 5–10 respiratory cycles, synchronized with the inspiratory pause. The radiographs were acquired using a Shimadzu portable radiography system in the anteroposterior supine position and randomly presented to two radiologists for reporting.

**Results:**

The mean age of the patients was 63.5±14 years. Overall, 43 (57.3%) patients were male, and 32 (42.7%) were female. Therapeutic interventions were performed for only 8% of cases with conventional CXR versus 21.3% of cases with recruited CXR; the difference was found to be statistically significant (P<0.05). The diagnostic efficacy of portable recruited CXR versus conventional portable CXR was 45% versus 18.6%. Also, the therapeutic efficacy of portable recruited CXR versus conventional portable CXR was 21.3% versus 8%.

**Conclusion:**

Portable recruited CXR seems to be a valuable diagnostic approach for clinical decision-making, with higher diagnostic and therapeutic efficacies in mechanically ventilated patients.

## INTRODUCTION

Portable chest radiography (CXR) is a common diagnostic procedure in mechanically ventilated patients. Factors, such as patient and apparatus positioning and exposure setup, may influence the clinical interpretation and decision-making due to their impact on image quality (1–3). Portable CXR is performed as either a daily routine CXR that has low diagnostic value or an on-demand radiographic procedure, known as restrictive CXR strategy, which is applied for various purposes, such as monitoring of the tracheal tube and central venous catheter or identifying complications of mechanical ventilation, such as ventilator-associated pneumonia (VAP) and pneumothorax (4– 6).

During portable CXR, the amount and pressure of air in the lungs are dependent on the time of radiography. Generally, it is difficult to acquire radiographs at the point of maximum inspiration, as any inspiratory pause is too short. Most errors during CXRs for ventilated patients are due to patient movement or inadequate air in the lungs (7). However, there is very little information available in the literature on synchronizing radiography with inspiration. In this regard, a study on 25 patients reported that synchronizing portable CXR with the end of inspiration improves most radiographs (7). Another study demonstrated that positive end-expiratory pressure (PEEP) is an effective parameter in CXR (8). However, to date, no research has established the maximum pulmonary volume by introducing a maximum tidal volume with an inspiratory pause during the procedure.

In some previous studies, the applied methods only involved the comparison of routine with non-routine radiographic strategies. Considering the diagnostic and therapeutic importance of CXR in intensive care unit (ICU) patients, besides inadequate research on synchronizing CXR with inspiration, in this study, we aimed to perform portable recruited CXR by maximizing the volume of inspiration to compare its diagnostic and therapeutic efficacies with conventional portable CXR.

## MATERIALS AND METHODS

This diagnostic accuracy study was performed to determine the diagnostic and therapeutic efficacies of portable CXR in intubated, mechanically ventilated patients, who were admitted to the ICU of Imam Khomeini Hospital in Sari, Iran, during 2013–2015. A total of 75 intubated patients were included in this study. For every patient, CXR was performed in two different settings. First, conventional portable CXR was performed, and then, the ventilator setting was adjusted as follows for acquiring recruited CXR: Tidal volume of 10–12 cc/kg up to inspiratory plateau pressure <35 cmH2O; inspiratory time of 2–3 seconds; 5–10 respiratory cycles to reach the maximum inspiratory volume; and performing portable CXR during an inspiratory pause.

A standard radiography apparatus was used for all patients. The radiographic technique included a beam energy of 75–85 kVp and exposure of 1 mA/s with no grid (7). The radiographs were acquired using a Shimadzu mobile radiography system in the anteroposterior supine position. Since both radiographs were acquired sequentially without any changes in the patient or device position, they had similar features, especially in terms of the amount of radiation, patient’s distance from the device, and the patient’s position (9). For all patients, CXR was requested by the attending ICU clinician.

Before the CXR procedures, demographic data, including age, gender, underlying diseases, Acute Physiology and Chronic Health Evaluation II (APACHE II) score, length of ICU stay, and ventilator device setting were collected. The radiographs were available to the patient’s physician, and the therapeutic approaches were documented. The radiographs were coded according to the table of random numbers and presented to two radiologists for reporting. The data related to radiography reports were also documented. The Ethics Committee and the Vice Chancellor of Mazandaran University of Medical Sciences approved this study.

Descriptive statistics were measured to analyze qualitative variables, and mean±SD was measured for age and other quantitative variables. Also, to describe qualitative variables, frequency tables, and percentages were measured. To analyze the data, the Chi-square test and Fisher’s exact test were used. In all statistical tests, a P-value of less than 0.05 was considered significant.

## RESULTS

The mean age of 75 mechanically ventilated patients in this study was 63.5±14 years (age range: 42–75 years). The study population included 43 (57.32%) males and 32 (42.7%) females, and the difference was not statistically significant (P>0.05). The mean APACHE II score of the patients at the beginning of the study was 15±7. The demographic data are briefly presented in Table 1.

**Table 1.** Demographic data of the studied patients.

**Variable**		**N (%)**
**Number**		75
**Gender**	Male	43(57/3%)
	Female	32(42/7%)
**Average of age (Year)**		63/5± 14
**Average of stay in ICU (Day)**		4/5± ¼
**APACH II score**		15± 7
	Medical	30(40%)
**The reason for admission in ICU**	General Surgery	20(26/6%)
	Cardiopulmonary Surgery	15(20%)
	Neurosurgery	6(8%)
	Other	4(5/3%)

The new findings and therapeutic interventions are shown in Table 2. The number of newly discovered radiological abnormalities on portable recruited CXR versus conventional CXR and therapeutic interventions was 34 versus 14 (P=0.045) and 16 versus 6 (P=0.034), respectively.

**Table 2.** New radiographic findings and therapeutic interventions in conventional vs. recruited portable CXRs

**Variables**	**Conventional Portable CXR**		**Recruited Portable CXR**	

	Diagnosis	Therapeutic intervention	Diagnosis	Therapeutic intervention
Atelectasis	0	0	2	0
Parenchyma infiltrates	0	0	4	2
pulmonary congestion	0	0	4	2
Pleural abnormality	0	0	4	2
Malposition of invasive devices	14	6	17	10
Others	0	0	3	0

**Figure 1.**
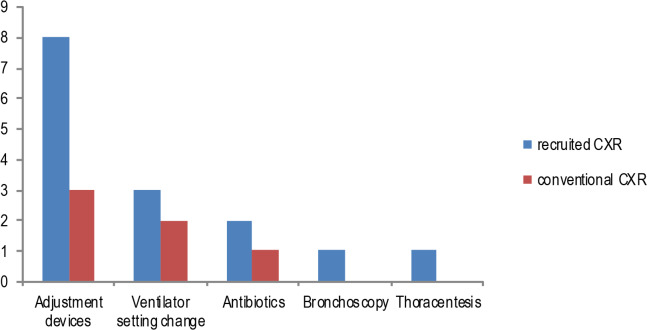
Therapeutic interventions based on CXR abnormalities in recruited and conventional portable CXRs

As shown in Table 3, the diagnostic efficacy of portable recruited CXR versus conventional portable CXR was 45% versus 18.6%. Also, the therapeutic efficacy of portable recruited CXR versus conventional portable CXR was 21.3% versus 8%. The therapeutic interventions for abnormalities detected on portable recruited CXR versus conventional portable CXR are shown in Figure 1.

**Table 3.** Diagnostic and therapeutic efficacies in recruited vs. conventional portable CXRs

**Variable**	**Conventional portable CXR**	**Recruited portable CXR**	**P**
**Total CXRs**	75	75	
**CXR with new changes (Diagnostic efficacy)**	14(18.6%)	34(45%)	0.045
**CXR with new changes + Therapeutic intervention (Therapeutic efficacy)**	6(8%)	16(21.3%)	0.034

## DISCUSSION

The present study showed that portable recruited CXR has significantly higher diagnostic and therapeutic efficacies in intubated, mechanically ventilated patients. Portable CXR, in addition to clinical and laboratory findings, plays an important role in the diagnosis and follow-up of VAP (10). Generally, VAP affects up to 20% of critically ill intubated patients and is associated with increased morbidity and mortality (11). However, due to the inaccuracy of clinical and radiological findings, it is difficult to diagnose and distinguish VAP from other respiratory diseases (12).

In addition to the challenges of radiographic diagnosis of VAP, interobserver variability is also high (11). As shown in the present study, it is clear that performing CXR with a higher quality, using a recruited maneuver, can be more effective in diagnosing and managing VAP. The ACR Appropriateness Criteria Expert Panel on Thoracic Imaging has recommended portable CXR for monitoring patients on mechanical ventilation, if clinically indicated, and also after the placement of tubes and catheters (6).

In previous studies, it has been shown that CXR has moderate accuracy in visualizing opacification without high sensitivity and specificity (13). In a recent study, which emphasized on the contributing factors for image acquisition and quality, and consequently, poor reliability of portable CXR interpretations, a novel method consisting of a variable attenuation plate and associated software was recommended to potentiate the improvement of patient care (14). Overall, our method of acquiring portable recruited CXR can be a reasonable alternative approach for increasing the diagnostic efficacy of portable CXR.

In a previous study, it was shown that portable CXR has a higher diagnostic accuracy for detecting the malpositioning of tubes and lines in comparison with parenchymal opacification (13). Our results showed that portable recruited CXR has higher diagnostic and therapeutic accuracies than conventional CXR, even for detecting the malpositioning of invasive devices. As shown in this study, pulmonary abnormalities, such as atelectasis, pulmonary infiltration, and pulmonary congestion were detected more accurately using our method. Our results also showed that portable recruited CXR has better diagnostic and therapeutic efficacies for pleural abnormalities.

In conclusion, portable recruited CXR can be a useful diagnostic imaging modality for clinical decision-making, with higher diagnostic and therapeutic efficacies in mechanically ventilated patients.
